# Using Knowledge Translation to Craft “Sticky” Social Media Health Messages That Provoke Interest, Raise Awareness, Impart Knowledge, and Inspire Change

**DOI:** 10.2196/mhealth.5987

**Published:** 2016-10-05

**Authors:** Sanchia Shibasaki, Karen Gardner, Beverly Sibthorpe

**Affiliations:** ^1^ ThinkThrough Consultancy Services Holland Park Australia; ^2^ Centre for Primary Health Care and Equity University of New South Wales Sydney Australia; ^3^ Consultancy Port Macquarie Australia

**Keywords:** knowledge translation, social media, Indigenous health, health promotion

## Abstract

**Background:**

In Australia, there is growing use of technology supported knowledge translation (KT) strategies such as social media and mobile apps in health promotion and in Indigenous health. However, little is known about how individuals use technologies and the evidence base for the impact of these health interventions on health behavior change is meager.

**Objective:**

The objective of our study was to examine how Facebook is used to promote health messages to Indigenous people and discuss how KT can support planning and implementing health messages to ensure chosen strategies are fit for the purpose and achieve impact.

**Methods:**

A desktop audit of health promotion campaigns on smoking prevention and cessation for Australian Indigenous people using Facebook was conducted.

**Results:**

Our audit identified 13 out of 21 eligible campaigns that used Facebook. Facebook pages with the highest number of likes (more than 5000) were linked to a website and to other social media applications and demonstrated stickiness characteristics by posting frequently (triggers and unexpected), recruiting sporting or public personalities to promote campaigns (social currency and public), recruiting Indigenous people from the local region (stories and emotion), and sharing stories and experiences based on real-life events (credible and practical value).

**Conclusions:**

KT planning may support campaigns to identify and select KT strategies that are best suited and well-aligned to the campaign’s goals, messages, and target audiences. KT planning can also help mitigate unforeseen and expected risks, reduce unwarranted costs and expenses, achieve goals, and limit the peer pressure of using strategies that may not be fit for purpose. One of the main challenges in using KT systems and processes involves coming to an adequate conceptualization of the KT process itself.

## Introduction

In Australia, the use of technology supported knowledge translation (KT) strategies like social media, mobile software apps, patient-mediated tools, and clinical decision support systems in health promotion and in Aboriginal and Torres Strait Islander (hereafter respectfully referred to as Indigenous) health is growing [1,2]. This corresponds with a growing use of social media among Australians in general, and in particular among Indigenous people, whose use of Facebook is 20% higher than the national average [3].

Despite this growing use, little is known about how individuals use technologies and evidence of the benefit and impact of these social media applications on health behavior change is meager. In their review of use in Indigenous populations, Brusse and colleagues found that the benefit and impact of social media applications was tentative and scattered, suggesting that producers of health promotion projects needed to obtain a thorough understanding about who engages with these strategies, why they engage, and how they engage [2]. The authors recommended further research in KT and implementation to better understand how to translate principles of commercial success in social media and mobile software into effective health promotion interventions and how to better integrate these methods into health research [2].

KT in health is defined as a “dynamic and iterative process that includes synthesis, dissemination, exchange, and ethically sound application of knowledge to improve health, provide more effective health services and products, and strengthen the health care system” [4]. Using KT strategies to support the design and deployment of health technologies is likely to increase their effectiveness and facilitate more efficient use of resources. However, Goering and colleagues suggest that whereas researchers and others are being encouraged to incorporate KT activities and strategies into their research applications, many are unclear about precisely what this means or how it should be assessed [5].

Three applications of KT, the Barwick KT planning Template [6] and the simple, unexpected, concrete, emotional, stories (SUCCESS) and social currency, triggers, emotion, public, practical value, stories (STEPPS) frameworks [7,8] are processes that have been developed to identify and shape KT strategies so they are fit for purpose for a particular context and a defined audience and for achieving a set of goals and impact. The Barwick Template incorporates a set of guiding questions and evidence-based checklists and refers to other KT models and frameworks that are associated with planning for implementation (eg, Consolidated Framework for Implementation Research, Knowledge to Action, Reach Effectiveness Adoption Implementation Maintenance RE-AIM) and for impact (eg, stickiness frameworks such as SUCCESS and STEPPS, and planning for evaluation eg, measurement indicators) [6]. The SUCCESS and STEPPS frameworks support planning for impact [7,8]. A health message or strategy should have impact such that it “catches on” or is understandable, memorable, and effective in changing thought or behavior. These characteristics are known as stickiness factors (Table 1) [7,8].

In this study, we examined how Facebook is used to promote health messages to Indigenous people on tobacco smoking prevention and cessation. We discuss how KT planning can support teams to plan, develop, and implement health messages to ensure chosen strategies are fit for purpose and designed to achieve impact. Although this study focussed on health promotion campaigns about tobacco use for Indigenous people, the outcomes are generalizable to other types of health campaigns and topics.

## Methods

### Data Collection

In 2015, a desktop audit was undertaken of the Indigenous HealthInfoNet health promotion resource database for tobacco campaigns published from 2005 to 2015. Campaigns were selected for inclusion in the study if they addressed smoking cessation and prevention, were nonpaper-based with an associated social media presence, and were audiovisual. Social media presence was confirmed by a search using Google Chrome (Google, Mountain View, CA, USA), Facebook (Facebook, Inc, Menlo Park, CA, USA), YouTube (YouTube, LLC, San Bruno, CA, USA), and Twitter (Twitter, Inc, San Francisco, CA, USA). Campaigns were excluded if they could not be retrieved, were duplicates, or also targeted a mainstream audience. A subset using Facebook were subjected to further analysis.

### Data Extraction and Analysis

Data about the campaign, the year and state in which it was developed, the producer, type of media strategy, number of likes, links to other websites, and average posts per month were entered into a Microsoft Excel database.

Campaigns were ranked from the highest number of likes to the least number of likes. A “like” indicates an appreciation, enjoyment, or support of the content posted on the Facebook page. The total number and mean number of posts per month were calculated for each Facebook page and pages were categorized into 2 groups: pages with more than 5000 likes and pages with less than 1800 likes (range is 35 to 11,000 likes). The number and type linkages between Facebook and Web-based applications such as websites and other social media applications (eg, Twitter and YouTube) were also analyzed.

The KT Planning Template and stickiness frameworks, SUCCESS and STEPPS, were then used to identify factors that may have contributed to the differences in overall likes, and to analyze key characteristics of interventions to assess the extent to which strategies are “fit for purpose” and to explain their uptake or impact.

### Ethics Approval

Ethics approval was not sought as the desktop audit collected data that were publicly available and freely accessible from public profiles on Facebook.

## Results

We identified 113 tobacco-related campaigns. Of these, 30 were selected based on our inclusion criteria. Of these, 6 were identified as duplicates and 3 were excluded because they were for Indigenous as well as mainstream audiences resulting in 21 campaigns for further examination (Table 2).

A range of social media applications were used in the 21 campaigns. The most popular were websites, YouTube, Facebook, and Twitter (Table 3). The most popular format to promote messages was videos.

Facebook pages with the highest number of likes (more than 5000) were linked to a website and to other social media applications. Linking social media applications and websites allows owners to consistently promote campaigns across all social media applications to ensure a broad range of audiences is captured and to “trigger” a reminder for audiences that use one or more applications.

Based on the average number of posts per month and posts' content, it appears pages with the highest number of likes (Deadly Choices, 11,000; Drug Info, 6829; Rockhole, 6304; Indigenous lung cancer ads, 5326) also shared similar stickiness characteristics such as social currency, triggers, emotion, public, stories, simple, credible, and practical value (Table 5).

**Table 1:** List of stickiness factors.

Characteristic	Short description
**Making ideas stick framework**	
Simple	Finding and delivering the core of message in a way that is so profound that a person could spend a lifetime learning to follow it.
Unexpected	Engaging people’s curiosity over long periods of time by systematically opening gaps in their knowledge and filling those gaps. Involves attracting a person’s attention (surprise) and holding that attention (interest).
Concrete	Helping people understand and remember messages through the use of concrete images such as the use of proverbs.
Credible	Ensuring messages carry their own credentials through the use of external (eg, an expert or authority figure) and internal credibility (eg, use of evidence and statistics).
Emotional	Messages that make people feel something by using the power of association, appealing to self-interest, and identify.
Stories	Stories can tell people how to act or how they can inspire (ie, give people the energy to act).
**Contagious framework**	
Social currency	People like to make a good impression, so products and ideas that make people look good are more likely to be shared.
Triggers	Triggers and cues lead people to talk, choose, and use. Social currency gets people talking. Triggers keep people talking. Top of the mind means tip of the tongue.
Emotion	Similar to making ideas stick framework—see above. Activating the right type of emotions is the key to transmission. When we care, we share.
Public	People are said to imitate one another. So if people can’t see what others are doing, they can’t imitate them. Making products and ideas popular means making them more publicly observable. If something is built to show, it’s built to grow.
Practical value	Practical value is about helping. Information that contributes to something being useful in terms of saving money, making people happier, or saving time is news you can use.
Stories	Similar to making ideas stick framework—see above. A narrative that people will want to share.

**Table 2:** Campaigns selected for further examination.

No.	Title	Year	Producer	State^a^	Format
1	Give up the smokes	2015	Bega Garnbirringu	WA	Video
2	My QuitBuddy	2015	Quit Now	National	Mobile app
3	Quit for you - quit for two	2014	Quit Now	National	Mobile app
4	Indigenous mothers talk	2014	Rural Health Channel	QLD	Video
5	Puyu paki (Don't smoke - give it up)	2014	Puntukurnu Aboriginal Medical Service	WA	Video
6	Breathe clearly, live healthy, quit smoking	2014	Mawarnkarra Health Service	WA	Video
7	Skinnyfish music health promotion videos	2013	Skinnyfish music	NT	Video
8	Indigenous lung cancer ads	2013	The Lung Foundation	National	Video
9	Rockhole	2013	Indigenous Hip Hop Projects	National	Video
10	Tomorrow's dream advertisement	2013	Aboriginal Health Council of Western Australia	WA	Video
11	Deadly Choices - smoking television commercial #1	2013	Deadly Choices	QLD	Video
12	Stickin it up the smokes: Ellie Lovegrove and Daniel Summer	2013	Lovegrove E. and Summer D.	SA	Video
13	Tobacco addiction story – English	2013	No Smokes	NT	Video
14	Smoking and pregnancy	2013	No Smokes	NT	Video
15	Quit Now online calculator	2013	Quit Now	National	Online calculator
16	Smoke-free homes and cars	2013	Aboriginal Tobacco Control Project	NSW	Video
17	Blow away the smokes: A guide to quitting cigarettes for Aboriginal and Torres Strait Islander people	2012	Baker F. and Gould G.	NSW	Video
18	Stay strong and healthy: Pregnancy resources for Aboriginal women	2012	NSW Ministry of Health	NSW	Mix
19	No durri for this Murri	2012	North Coast Aboriginal Corporation for Community Health	QLD	Video
20	VACCHO^b^ World No Tobacco Day	2011	Gallagher, J. Victorian Aboriginal Community Controlled Health Organisation	VIC	Video
21	DrugInfo	2011	Australian Drug Foundation	National	Website

^a^NSW: New South Wales; NT: Northern Territory; QLD: Queensland; SA: South Australia; VIC: Victoria; WA: Western Australia.

^b^VACCHO: Victorian Aboriginal Community Controlled Health Organisation.

**Table 3:** Social media applications used by 21 smoking cessation and prevention campaigns for Indigenous people.

Social media application	Number of tobacco prevention and cessation campaigns
Website	20
YouTube	18
Facebook	13
Twitter	11
Mobile app	2
Other (SoundCloud, Pinterest, Vimeo)	5

**Table 4:** Ranking of Facebook tobacco prevention and cessation campaigns.

No.	Title of health promotion program	Year	Producer	State^a^	Type	Likes	Linking	Average number of posts per month
1	Deadly Choices (smoking television commercial #1) [9]	2013	Deadly Choices	QLD	Video	11,000	Facebook to embedded videos and website Website to Facebook and Twitter	13
2	DrugInfo [10]	2011	Australian Drug Foundation	National	Website	6829	Facebook to Website Website to Twitter Twitter to Website	13
3	Rockhole [11]	2013	Indigenous Hip Hop Projects	National	Video	6304	Facebook to embedded videos Website to videos	15
4	Indigenous lung cancer ads [12]	2013	The Lung Foundation	National	Video	5326	Website to Facebook and videos	12
5	Tobacco addiction story - English [13]	2013	No Smokes	NT	Video	1741	Facebook to Twitter and videos (Youtube and Vimeo) Twitter to Website and videos	8
6	Smoking and pregnancy [13]	2013	No Smokes	NT	Video	1741	Facebook to Twitter and videos (Youtube and Vimeo) Twitter to Website and videos	8
7	VACCHO World No Tobacco Day [14]	2011	Victorian Aboriginal Community Controlled Health Organisation	VIC	Video	989	Facebook to Website and videos (Youtube) Twitter to Website Website to Facebook, Twitter, Youtube, and Soundcloud (audio)	5
8	Stay strong and healthy: Pregnancy resources for Aboriginal women [15]	2012	NSW Ministry of Health	NSW	Mix	831	Facebook to Website	4
9	Smoke-free homes and cars (Facebook- I quit because)[16]	2013	Aboriginal Tobacco Control Project	NSW	Video	466	Facebook to Website and embedded videos Website to videos	12
10	Blow away the smokes: A guide to quitting cigarettes for Aboriginal and Torres Strait Islander people [17]	2012	Baker, F. and Gould, G.	NSW	Video	55	Website to Facebook and videos (vimeo)	0
11	Give up the smokes [18]	2015	Bega Garnbirringu	WA	Video	52	Website to Youtube	0.1
12	Tomorrow's dream advert [19]	2013	Aboriginal Health Council of Western Australia	WA	Video	42	Facebook to Website Website to Facebook, Twitter, Google Plus, PinInterest, Soundcloud	0.1
13	Breathe clearly, live healthy, quit smoking [20]	2014	Mawarnkarra Health Service	WA	Video	35	Twitter to Facebook	0

^a^NSW: New South Wales; NT: Northern Territory; QLD: Queensland; SA: South Australia; VIC: Victoria; WA: Western Australia.

**Table 5:** Stickiness factors associated with Facebook pages with likes more than 1000.

Stickiness factors	Stickiness factors from Facebook pages with high number of likes more than 5000
Social currency	Demonstrating the adoption of current trends or the rollout of new initiatives such as tackling smoking Promoting and sharing photos and video posts that showed people with a known sporting identity at a health promotion event where participants received a t-shirt or some other incentive
Triggers	Routine and regular posts of photos, videos, and information at different times of the day about upcoming campaigns Linking of social media applications to allow sharing of posts between platforms, thereby providing regular reminders
Emotion	Posting stories about personal life journeys and experiences such as quit smoking stories Use of music and dance to promote health promotion messages
Public	All profiles were public profiles - free to access Using tags to share posts with other Facebook profiles Linking of social media applications to allow sharing of posts between platforms such as YouTube and Facebook
Stories	Posting videos of ex-smokers sharing their stories about quitting smoking or about the death of a loved one due to lung cancer
Credible	Stories and videos were from an individual’s personal life journey and experience Advertisements promoted statistics about smoking and lung cancer
Practical value	Posting of videos about how to stay fit and healthy Posting of educational messages

For example, the Deadly Choices Facebook page demonstrated the following characteristics:

- Simple: Core message promoted consistently through written posts, photos, and videos.
- Unexpected: Frequently promoted competitions, free giveaways, and meet and greets.

- Triggers: The page frequently posted messages at different times of the day. It used different forms of media such as text, photos, and videos. The page was also linked to other social media platforms to allow sharing of posts to Twitter and YouTube.
- Social Currency and Public: The campaign appeared to have recruited known sporting and public personalities to promote campaigns. The page also posted photos and videos of people with sporting and public personalities at various health promotion campaigns.
- Stories, Emotion, and Credibility: The page posted or linked to videos of Indigenous people telling their stories about their quit smoking journey. There were also good news stories about the benefits of a healthy lifestyle through diet and exercise.
- Practical Value: Provided information about how to stay fit and healthy or how to cease smoking and get fit and healthy.

## Discussion

### Principal Findings

Using social media like Facebook has certain appeal: the potential to reach large numbers of people with ease of set up and at a minimum cost. There appears to be a perception that few resources, if any, are required to make health messages “sticky.”

However, this type of thinking is deceiving. The findings from this study show that the use of social media applications like Facebook do not guarantee that a campaign will have the desired impact and reach, such as through high numbers of likes, shares, and comments. Making health messages sticky through social media such as Facebook requires, at a minimum, content to be sourced and translated into a format that incorporates stickiness characteristics, routine posts that maintain page currency, routine monitoring and evaluating to assess impact and effectiveness, and a skilled and experienced workforce. Workforce expenses are the “hidden costs” of social media applications.

In addition to social media, there are several other strategies to translate knowledge. For example, strategies like knowledge brokers, champions, media campaigns, and pop-up stalls may be more suitable for campaigns that wish to provoke interest and raise awareness in groups that do not use social media or have limited access to the Internet. These strategies may have been more suitable for health promotion campaigns that received less than 500 Facebook likes. However, strategies like financial incentives, new policies, patient education sessions, and communities of practice could be used in combination with social media applications to impart knowledge and inspire change. For example, the Deadly Choices page promotes Indigenous designed t-shirts that are given to individuals who complete the annual health assessment. Once strategies are identified, the next step makes them sticky.

KT planning can be used as a tool to support individuals and teams to craft the delivery of health messages so that they are best suited and well-aligned to the campaigns’ goals, messages, and target audiences. KT planning has clear potential to help mitigate unforeseen and expected risks, reduce unwarranted costs and expenses, achieve goals, and limit the peer pressure of using strategies that may not be fit for purpose.

### Limitations

Due to privacy requirements, the desktop review did not have access to Facebook metrics, such as Page Insights, to measure the reach and uptake of posts. Page insights provide information about the number of people to whom a post has reached; who have clicked on a post; liked, commented, or shared a post; or viewed a video. This level of analysis would be important in any evaluation of specific campaigns.

### Future Implications

The uptake and use of frameworks and practices like KT planning will take time and will undoubtedly face challenges and barriers. The main challenges are conceptualizing KT and then applying it effectively to the local context [5]. Until this and other challenges (eg, limited organisational KT capacity, limited to access to KT workforce) are addressed, we will continue to use systems and processes that are familiar or easy to use but may be ineffective or have variable uptake and impact.

## Abbreviations

KT: knowledge translation
STEPPS: social currency, triggers, emotion, public, practical value, stories
SUCCESS: simple, unexpected, concrete, emotional, stories
VACCHO: Victorian Aboriginal Community Controlled Health Organisation
